# Adsorption-Hydration Sequence Method for Methane Storage in Porous Material Slurry

**DOI:** 10.3389/fchem.2020.00294

**Published:** 2020-04-21

**Authors:** Jun-Li Chen, Peng Xiao, De-Xin Zhang, Guang-Jin Chen, Chang-Yu Sun, Qing-Lan Ma, Ming-Ke Yang, En-Bao Zou

**Affiliations:** State Key Laboratory of Heavy Oil Processing, China University of Petroleum, Beijing, China

**Keywords:** methane hydrate, formation improvement, ZIF-8, slurry, gas storage

## Abstract

Porous materials are deemed to be capable for promoting hydrate formation, while for the purpose of hydrate-based gas storage, those systems containing porous materials often cannot meet the requirement of high storage density. To increase the storage density, an adsorption-hydration sequence method was designed and systematically examined in this study. Methane storage and release in ZIF-8 slurries and fixed beds were investigated. The ZIF-8 retained 98.62%, while the activated carbon lost 62.17% of their adsorption capacities in slurry. In ZIF-8 fixed beds, methane storage density of 127.41 V/V_bed_ was acquired, while the gas loss during depressurization accounted for 21.50% of the gas uptake. In the ZIF-8 slurry, the storage density was effectively increased with the adsorption-hydration sequence method, and the gas loss during depressurization was much smaller than that in fixed beds. In the slurry, the gas uptake and gas loss decreased with the decrease of the chilling temperature. The largest gas uptake and storage density of 78.84 mmol and 133.59 V/V_bed_ were acquired in the slurry with ZIF-8 content of 40 wt.% at 268.15 K, meanwhile, the gas loss just accounted for 14.04% of the gas uptake. Self-preservation effect was observed in the slurry, and the temperature for the slowest gas release was found to be 263.15 K, while the release ratio at 10 h reached to 43.42%. By increasing the back pressure, the gas release rate could be effectively controlled. The gas release ratio at 1.1 MPa at 10 h was just 11.08%. The results showed that the application of adsorption-hydration sequence method in ZIF-8 slurry is a prospective manner for gas transportation.

## Introduction

Gas hydrates are non-stoichiometric crystalline compounds formed when gas molecules with suitable size are trapped in polyhedral cavities of hydrogen-bonded water molecules under low temperature and high pressure. Natural gas hydrate extensively exists in the permafrost and the marine sediments and it is considered the largest hydrocarbon resource on earth. Gas hydrates are also being studied as alternative methods for industrial gas separation and carbon capture (Xu et al., 2012; Cai et al., 2017), water purification (Song et al., 2016; He et al., 2018; Dong et al., 2019), cold storage (Dufour et al., 2017) and food industry (Li et al., 2015). Meanwhile, because of its large gas storage capacity (up to 180 volume of gas per volume of hydrate) (Sloan, 2003), nonexplosive nature and mild storage condition (−5°C at 1 atm) (Stern et al., 2001), gas hydrate is also a potential way to transport natural gas. For the small gas fields and sporadic transportation, transporting natural gas via hydrate is superior to LNG and pipeline because of the investment flexibility, and it could displace the CNG because of its safety. However, there exists two impediments in the practical application of hydrate-based gas transportation: the slow formation kinetics and the low storage capacity.

In order to solve these two fundamental problems, chemical methods of adding kinetic (Zhong and Rogers, 2000; Wang et al., 2015) or thermodynamic additives (Kim et al., 2015; Liao et al., 2015) and physical methods including stirring (Hao et al., 2007; Veluswamy et al., 2017), bubbling (Luo et al., 2007; Lv et al., 2012), spraying (Fukumoto et al., 2001; Fujita et al., 2009) have been studied, and the hydrate formation could be accelerated by these methods to varying degrees. However, some drawbacks hinder the practical application of these methods: the typical kinetic additive, sodium dodecyl sulfate (SDS), triggers the capillary effect so that give fast formation kinetics (Gayet et al., 2005), at the same time, it leads to climbing wall growth and porous morphology (Zhong and Rogers, 2000; Gayet et al., 2005; Mandal and Laik, 2008), which would remarkably reduce the apparent storage density of the hydrate; the thermodynamic additives occupy some cages of hydrate (Kim et al., 2015), decreasing the theoretical storage capacity; for physical methods, the viscosity increase accompanied by aggregation of hydrate particles results in high energy consumption of stirring (Fidel-Dufour et al., 2006; Mori, 2015); the hard-to-broken hydrate shells occupy the gas space, hindering the further formation in bubbling column (Luo et al., 2007); the heat transfer restricts the formation rate in spraying reactor (Matsuda et al., 2006).

Porous materials also have been used for improving hydrate formation. The typical application includes forming fixed bed and particle suspension. In water-contained fixed bed, the extensive contact area on packing material intensifies the hydrate formation. Porous media including silica sand (Linga et al., 2012; Babu et al., 2013; Yang et al., 2016), silica gel (Dicharry et al., 2013; Kumar et al., 2015), glass beads (Yang et al., 2015) have been proved to be effective in improving hydrate formation. Linga et al. (2012) studied methane hydrate formation in a fixed bed filled with sand and found that the hydrate formation was much faster than that in a stirred reactor, and the gas uptake reached to 193.13 V/V_water_. Some research revealed that porous materials including activated carbon (Yan et al., 2005; Zhou et al., 2005; Siangsai et al., 2015) and MOFs (Mu et al., 2012; Casco et al., 2016) also have significant effect on hydrate formation. Babu et al. (2013) studied the morphology of methane hydrate formation in activated carbon. The hydrate formed in the interstitial pore space between the particles, thus, they concluded that the pore space plays an important role in hydrate formation. Porous materials could promote hydrate formation, in turn, hydrate formation could increase the gas storage capacity of the fixed bed. Zhou et al. (2005) found that the sorption amount of methane on wet activated carbon increased with the increase of water content, and when the water content *R*_*w*_ = 2.92, the sorption amount was 3.75 times higher than that on dry carbon because of the hydrate formation. Similarly, Yan et al. (2005) acquired a gas uptake of 140 V/V_bed_ in moist carbon. Mu et al. (2012) suggested that by adding some water in ZIF-8 fixed bed, the storage capacity could be raised by more than 56%. All of these fixed beds are in favor of hydrate formation, however, from the perspective of gas transportation, a contradiction exists between the hydrate formation and storage density. Generally, hydrate formation is well promoted only when water content is small. Chari et al. (2013a) measured the storage capacity of a silica-water system with water content from 20 to 1 g/g_silica_, and found that the methane conversion monotonically increased from 6.14 to 67.82%, which suggested that the small water content is in favor of hydrate formation. However, the small water content could result three problems: (1) the gas fixed on porous materials by Van Der Waals force is easy to desorb during depressurization; (2) a large amount of porous media in fixed bed increases the apparent volume, so that decreases the storage density; (3) the scattered and small hydrate particles are easy to dissociate (Takeya et al., 2005). Therefore, to increase the feasibility of gas storage and transportation via hydrate formation in fixed bed, the water content needs to be increased. However, increasing water content would cause some problems.

Some studies suggest that in water dominated systems like suspension and slurry, solid particles have certain positive effects on hydrate formation. Zhou et al. (2014) found that by adding 0.4% graphite nanoparticle into the water, the induction time of CO_2_ hydrate decreased by 80.8% and the CO_2_ consumption increased by 12.8%. Kim et al. (2011) indicated that the multi-walled carbon nanotubes could accelerate hydrate formation. Pasieka et al. (2013) found that both the hydrophobic and hydrophilic multi-wall carbon nanotubes enhanced the hydrate formation. Similarly, porous materials also promote hydrate formation in suspension. Govindaraj et al. (2015) compared the effects of activated carbon and nano-silica on methane hydrate formation in suspension, and concluded that the effect of activated carbon is more pronounced. Wang et al. (2012) found that 0.01% ZIF-61 could accelerate the nucleation of tetrahydrofuran hydrate. Casco et al. (2016) compared the effects of MOFs with hydrophobic nature (ZIF-8) and hydrophilic nature [MIL-100(Fe)] on methane hydrate formation, and they found that the ZIF-8 caused a higher hydrate yield.

Hydrate formation in these suspensions could be enhanced in some extent, however, the solid particles mainly act as the nucleation center. In the process of transition from fixed bed to suspension by increasing water content, once the water content exceeds a certain value, the storage capacity of the fixed bed decreases rapidly (Yan et al., 2005), resulting low gas storage capacity in suspension. The highest storage capacity in the suspension of multi-walled carbon nanotubes was about 11.94 V/V_w_ at 10 h in Kim et al. (2011) work. The average water conversion was only 19.3% after 24 h in Govindaraj et al. (2015) study. Thus, simply increasing the water content could not resolve the contradiction between hydrate formation and storage density.

Besides the hydrate formation kinetics and gas storage density, hydrate dissociation is also a key factor to assess if the system is suitable for gas storage and transportation. Generally, hydrate dissociation rate decreases with the increase of pressure (Circone et al., 2004), while it does not simply decreases with the decrease of temperature because of the self-preservation effect (Stern et al., 2003), which could create a trough on the curve of dissociation rate near the ice point, and it is considered to be the basis of hydrate transportation. Hydrate dissociation also relates to other factors. Takeya et al. (2005) indicated that larger hydrate particles are in favor of decreasing hydrate dissociation. Liang et al. (2005) found that about 6% of hydrate dissociated in 10 h at 267.4 K and the presence of activated carbon increased the dissociation. Compared with the pure water, additive like SDS (Lin et al., 2004), treated nano-particles (Wang et al., 2016), Salt (Mimachi et al., 2016) also would increase the dissociation rate.

In order to resolve the contradiction between the hydrate formation and the gas storage density, some attempts have been made in our previous study (Xiao et al., 2019), and both the hydrate formation and storage density were improved in the fixed bed with high water content. However, the highest storage density was only 111.75 V/V_bed_, and the gas release was too fast for gas transportation. In the current study, we are trying to find a manner to promote hydrate formation in slurry, so that to acquire high gas storage density and slow gas release rate simultaneously.

## Experimental Section

### Material

Methane with a purity of 99.99% was supplied by Beijing Haipu Gas Co., Ltd. Double distilled water was prepared in our laboratory. ZIF-8 was synthesized in our laboratory. Activated carbon with particle size of 100 mesh was purchased from Sigma-Aldrich.

### Apparatus

**Figure 1:**
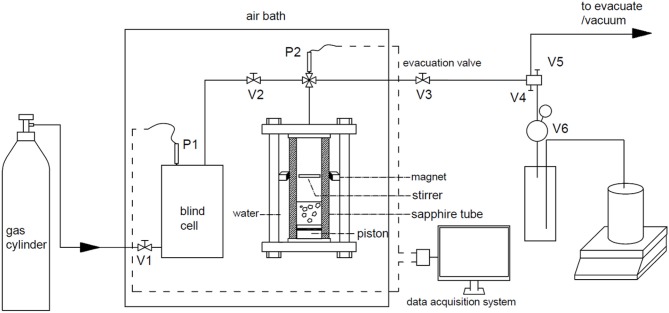
Schematic diagram of the experimental apparatus.

The setup used in this study is shown in Figure 1. The main parts of the apparatus are a stainless-steel blind cell and a sapphire cell. The effective volume of the blind cell and the tubes connected on is 130.23 cm^3^, and that of the sapphire cell is 61.90 cm^3^. The evacuation tube is separated into two lines by V4 and V5. A water displacement device is connected on the V5 through a back-pressure valve V6, which is used to keep the pressure of sapphire cell constant in dissociation experiments, and the range of it is 0 ~ 1.6 MPa. The displaced water is weighed with a balance and the mass data are recorded by the computer every 5 s. The blind cell and the sapphire cell are installed in an air bath to keep temperature constant, and the pressure of them are determined with two sensors with accuracy of 0.2% in the range of 0 ~ 20 MPa.

### Experimental Procedure

**Figure 2:**
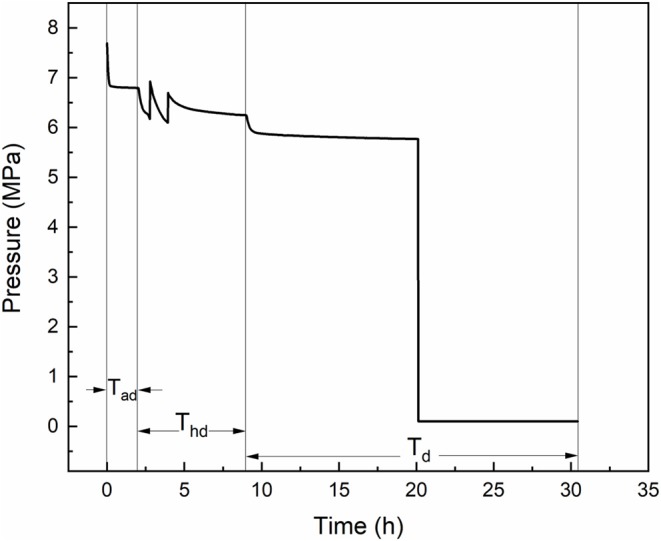
Typical pressure change in the experiment (*P*_*b, d*_ = 0.1 MPa).

The formation experiment of methane hydrate followed an adsorption-hydration method. Ten gram of porous material/water mixture was loaded in the sapphire cell. When the low water content was adopted (fixed bed), the mixture was compacted with a PTFE rod. The sapphire cell was installed in the air bath, then the stirrer was switched on if the mixture is slurry state. The blind cell was purged by charging methane and vacuuming three times, then it was pressured to 11 MPa with methane. The sapphire cell was vacuumed to 0.003 MPa to desorb the gas adsorbed on the porous material. The temperature of the air bath was set to 293.15 K (*T*_*ad*_) at first—such high temperature was chosen to avoid the hydrate formation during fast adsorption period. When the pressure of the blind cell kept constant for 30 min, the injection valve V2 was opened and the sapphire cell was charged by methane to about 7.8 MPa, then gas adsorption began. The high pressure could provide fast adsorption in the beginning and remain large driving force when the temperature decreases to the hydrate formation region. After the system reached adsorption equilibrium, the temperature of the air bath was set to 278.15 K (*T*_*hd*_) to allow hydrate formation and avoid the water freeze, once the hydrate formed the stirrer was switched off, and methane hydrate formed quiescently during the cooling process. When the decrease rate of reactor pressure lower than 1 kPa per min, the temperature (chilling temperature, *T*_*d*_) was set to the values below ice point to freeze the hydrate, porous material and unconverted water, so that to retard the gas release. The chilling temperature was between 268.15 and 259.15 K, because the hydrate dissociation in pure water has been proved to be the slowest at 268.15 K (Stern et al., 2001). The chilling procedure lasted for at least 3 h, then the temperature in the reactor was believed to have reached the set value. The valves V3 and V4 were opened and the sapphire was rapidly depressurized to desired value *P*_*b, d*_, then the V4 was closed and V5 and V6 were opened, and water in the tank was squeezed out by the released gas. The mass of the displaced water was measured by an online balance and was recorded for every 5 s. During the gas release procedure, the water tank was kept raising to ensure the water level was of the same height as the extremity of the drain pipe. The typical pressure curve is presented in Figure 2.

### Calculation of the Methane Storage Capacity and Release Rate

The gas storage density was indicated by *S*_*b*_, and it is calculated by

(1) $$\begin{array}{r} S_{b}=\frac{22.4\times 10^{-3}N}{V_{bed}} \end{array}$$

The *V*_*bed*_ represents the apparent volume of the frozen fixed bed, which is composed of hydrate, ice and porous material. It was calculated by

(2) $$\begin{array}{r} V_{bed}=V_{0}+0.25V_{w} \end{array}$$

where the *V*_0_ refers to the initial volume of the slurry or the fixed bed, and it was measured directly. The *V*_*w*_ refers to the volume of water. The *N* in equation (1) refers to the number of moles of gas fixed in the frozen bed, and it was calculated by

(3) $$\begin{array}{r} N=\frac{P_{a,i}V_{a}}{Z_{a,i}RT_{ad}}-\frac{P_{a,d}V_{a}}{Z_{a,d}RT_{hd}}-\frac{P_{b,d}V_{gas}}{Z_{gas}RT_{hd}} \end{array}$$

where the *P* and *Z* represent pressure and compressibility factor, respectively. The compressibility factor *Z* was calculated by Benedict-Webb-Rubin equation. The subscript *a* and *b* represent the blind cell and sapphire cell, respectively. Subscript *i* refers to the time point of gas injection from blind cell to sapphire, and the subscript *d* refers to the time point of depressurizing the sapphire cell to dissociation pressure. *V*_*a*_ refers to the effective volume of blind cell. *V*_*gas*_ represents the volume of free gas in the sapphire cell, and it was calculated by

(4) $$\begin{array}{r} V_{gas}=V_{b}-m\upsilon \left(1-\varepsilon \right)-1.25V_{w} \end{array}$$

where the *V*_*b*_ is the effective volume of the sapphire cell. *m* represents the mass of the porous material, and υ refers to the packing density of the compacted porous material, m^3^/g. ε is the porosity of the compacted material, and it was measured to 0.38 in this study.

In the gas release experiment, the mole number of the collected gas *N*_cg_ was calculated by

(5) $$\begin{array}{r} N_{cg}=\frac{m_{wc}}{\rho_{w}}\times \frac{273.15}{T_{env}}\times \frac{1}{22.4\times 10^{3}} \end{array}$$

where the *m*_*wc*_ is the mass of water displaced by the released gas, and the ρ_*w*_ represents the density of water, g/cm^3^. The *T*_*env*_ is the ambient temperature.

When depressurizing the reactor to desired dissociation pressure, some gas that had already fixed in the frozen bed escaped into the environment, and the escaped gas is called “gas loss” in this study. The number of moles of the gas loss was calculated by

(6) $$\begin{array}{r} \Delta N=N-N_{cg} \end{array}$$

In the gas release stage, the release ratio *R*_*r*_ at time *t* is calculated by

(7) $$\begin{array}{r} R_{r,t}=\frac{N_{cg,t}}{N_{cg}} \end{array}$$

where *N*_*cg, t*_ refers to the mole number of collected gas at time *t*. The moment that the pressure of the reactor decreased to desired value was set to time *0*.

## Results and Discussion

### Methane Storage Capacity

**Figure 3:**
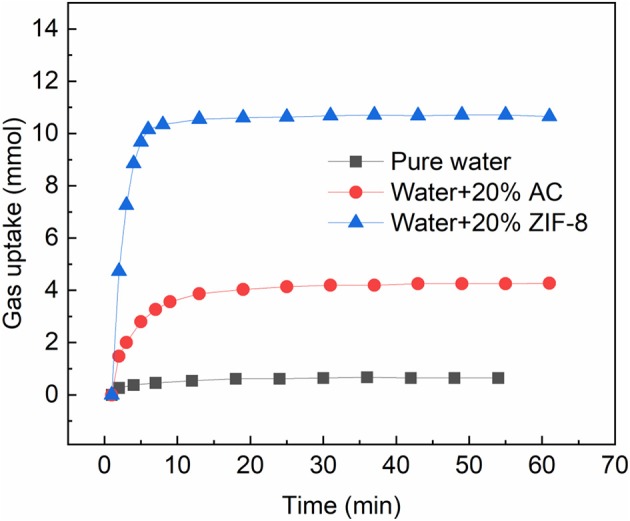
Gas uptake of methane in ZIF-8 and activated carbon slurries at 293.15 K and 6.0 MPa.

In the porous material contained hydrate formation system, both the gas adsorption and hydration contribute to the gas uptake. Though the existence of water weakens the gas adsorption, the gas adsorption remains an important factor for gas storage. It not only concerns the adsorption capacity, but also affects the hydration: adsorbed gas could be released from the porous material as pressure decrease caused by hydrate formation, and supplies gas from inside of the water. Figure 3 compares the adsorption capacity of methane on a hydrophilic material (activated carbon) and a hydrophobic material (ZIF-8) at 293.15 K and 6.0 ± 0.2 MPa in slurry. The mass of the slurries and water were 10 g, and the mass ratios of the porous material to slurry were 20 wt.%. As shown, only 0.64 mmol of methane was absorbed in the water. The methane uptake in the ZIF-8 slurry and activated carbon slurry were 10.65 and 4.28 mmol, respectively, which were obviously higher than that in pure water. The adsorption capacities of methane on dry ZIF-8 and activated carbon were 5.14 and 4.98 mmol/g at 293.15 K and 6.0 MPa (measured with a RUBOTHERM Gravimetric Sorption Analyzer), respectively. Compared with dry material, the activated carbon lost almost 62.17% of the adsorption capacity in the slurry, while ZIF-8 retained 98.62% of adsorption capacity. Meanwhile, the ZIF-8 slurry reached the adsorption equilibrium within 8 min, which was obviously faster than the activated carbon slurry. The higher adsorption capacity and faster adsorption rate makes it a better porous material to form slurry. It could adsorb more gas before being frozen in the hydrate/ice, so that increase the storage capacity of the slurry. In the following experiments, the ZIF-8 was chosen as the porous particle in the slurry.

The results of hydrate formation and gas release experiments in ZIF-8 slurries/fixed beds are listed in Table 1. Both the adsorbed and hydrated gas are included in the “gas uptake” in the table because there was no a distinct boundary between the adsorption and hydration process as the adsorption was a dynamic process affected both by decreasing temperature and pressure. The dynamic process could be described as: The system reached adsorption equilibrium at 293.15 K at first, then the temperature was adjusted to 278.15 K, more gas was adsorbed on the porous materials because the gas adsorption is more pronounced at lower temperature. When hydrate started to form in the slurry, there existed a dramatic pressure drop, the already-existed adsorption equilibrium was broken, and some gas was released from the porous materials. When the chilling process was started, some gas would be re-adsorbed on porous materials, and the re-adsorption could be affected by the freeze of the fixed bed. The “collected gas” refers to the recovered gas from the hydrate and the ZIF-8, and it was calculated based on the amount of the displaced water and the environment temperature. The “gas loss” is the amount difference between the gas uptake and the collected gas, and it reflects the gas release during depressurization. The “apparent bed volume” is the bulk volume of the mixture of ZIF-8, ice and hydrate before depressurizing the reactor to desired pressure.

**Table 1:** Experimental results of methane uptake and release in ZIF-8 slurries and fixed beds.

**No**.	***T_**d**_*(K)**	***P_**b, d**_*(MPa)**	**Solid content (wt.%)**	**Apparent bed volume (cm^**3**^)**	**Gas uptake (mmol)**	**Storage density (V/V_**bed**_)**	**Collected gas (mmol)**	**Gas loss (mmol)**
1	263.15	0.1	20	13.65	51.01	83.72	48.64	2.37
2	263.15	0.1	30	13.47	64.44	107.16	57.12	7.32
3	263.15	0.1	40	13.22	63.45	107.51	53.59	9.86
4	263.15	–	60	13.70	18.55	30.33	–	–
5	263.15	0.1	70	14.17	80.60	127.41	63.27	17.33
6	263.15	0.1	100	21.27	75.89	79.92	53.35	22.54
7	268.15	0.1	40	13.22	78.84	133.59	67.77	11.07
8	265.15	0.1	40	13.22	73.80	125.05	62.29	11.51
9	261.15	0.1	40	13.22	61.34	103.93	55.63	5.71
10	259.15	0.1	40	13.22	58.53	99.17	55.86	2.67
11	263.15	0.5	40	13.22	67.20	113.87	59.42	7.78
12	263.15	0.7	40	13.22	63.98	108.41	56.79	7.19
13	263.15	0.9	40	13.22	65.16	100.40	61.51	3.65
14	263.15	1.1	40	13.22	62.99	106.73	58.21	4.78

In runs 1 ~ 6, the gas storage capacity in ZIF-8 slurry/fixed bed with different solid contents were investigated. The mixture of ZIF-8 and water was in slurry form when the solid content was lower than 40 wt.%, and it was in fixed bed form when the solid content was higher than 60 wt.%. As shown, the apparent bed volume did not monotonously increase with the increase of solid content though the ZIF-8 has a bigger bulk volume than water under the same mass. It decreased with the increase of solid content at first and reached the smallest volume of 13.22 cm^3^ at solid content of 40 wt.%, then it increased with the increase of solid content. This phenomenon has been described by Mu et al., and they thought it was caused by the effect of water on ZIF-8 (Mu et al., 2012).

In the slurries (runs 1~3), the stirrer was switched off once the hydrate appeared in the slurry, hence the hydrate formation was actually conducted under quiescent condition. Generally, when hydrate quiescently forms in a water dominated system, a rigid hydrate film would appear at the interface between water and gas, resulting low water conversion. In the fixed beds packed with hydrophilic materials and saturated with high water cut, the gas storage capacity also is very small because the adsorption was weakened significantly by the water, meanwhile, the conversion of water to hydrate is hindered by the large water content (Yan et al., 2005). However, in the runs 1 ~ 3, high storage capacities were achieved even in slurry, and when the solid content was 40 wt.%, the storage density reached to 107.51 V/V_bed_. In many other researches that use porous materials to improve hydrate formation, the gas storage capacity was unsatisfactory: Kim et al. acquired the highest storage capacity of 13.44 V/V_water_ when 0.004 wt.% of multi-walled carbon nanotubes was added into water (Kim et al., 2011); Govindaraj et al. (2015) investigated the kinetics of hydrate formation in the activated carbon suspension (1.0 wt.%) at 275.15 K and 8 MPa and the highest gas storage capacity was 20.3 mmol/mol of water (25.26 V/V_water_) at 24 h Chari et al. (2013b)acquired a storage capacity of 91.72 V/V_water_ in nano silica suspensions (12.5 wt.%). The small gas storage capacity in these reports perhaps are mainly because the small dosage of the solid particles used in the suspension. In addition, Govindaraj et al. (2015) suggested that the hydrate formation was more favorable at higher particle concentration, thus Chari et al. (2013b) acquired the higher storage capacity than the other two research, and when they further increased the solid content to 25.0 wt.% (fixed bed), they acquired a much high storage capacity of 190.40 V/V_water_. Compared with the hydrate formation from suspension/slurry in those works, the storage capacity in ZIF-8 slurry in this work was higher. The high solid content used in this work is one of the reasons for high storage density in the slurry, however, the hydrophobicity nature of the ZIF-8 cannot be neglected either. The effect of the hydrophobicity of ZIF-8 on the storage density could be explained as: the adsorption capacity of ZIF-8 was retained in the slurry because of the hydrophobicity; large amount of gas was adsorbed on ZIF-8 particles, and some of it desorbed during the hydrate formation, providing gas from inside of the slurry, which could alleviate the problem that the hydrate forms slowly in slurry.

In the fixed beds (runs 4~6), no hydrate formation was observed at solid content of 60 wt.%. This was because the fixed bed was 100% saturated by the water, while the experiment was conducted quiescently. In such situation, the gas uptake of 18.55 mmol was almost contributed by adsorption. When the solid content increased to 70 wt.%, the water dispersion was improved in the fixed bed, and the gas uptake and storage density reached to 80.60 mmol and 127.41 V/V_bed_, respectively, which were the highest in runs 1 ~ 6. Compared with the solid content of 60 wt.%, the increased gas uptake was found to be mainly caused by hydrate formation in the fixed with solid content of 70 wt.%. In the dry bed, the gas uptake was slightly lower than that of the bed with solid content of 70 wt.%, however, the storage density was much lower because of the much bigger bulk volume of the dry ZIF-8.

By comparing the slurries and the fixed beds, it could be found that the highest storage density was acquired in the fixed bed with solid content of 70 wt.%. However, this does not mean that the fixed bed with small water content is the best solution for gas storage and transportation. In gas transportation, a low-pressure process could effectively decrease the potential risks and the equipment investment, while when depressurizing the system to a low pressure, some gas that have been fixed already could escape with the free gas. In runs 1~6, the gas released monotonously increased with the increase of solid content. It was only 2.37 mmol in the slurry with solid content of 20 wt.%, while it reached to 22.54 mmol in dry bed, which accounted for 29.70% of the gas uptake. This was because in the fixed beds with high solid contents, a big part of the gas was fixed by adsorption, which was maintained by Van der Waals force, and the adsorbate was easy to be released when the adsorption equilibrium was broken.

As discussed above, satisfactory storage density could be acquired in slurry and the gas loss was much lower than that in the fixed bed, this suggests that the ZIF-8 slurry could be used for gas storage and transportation by following the adsorption-hydration sequence method. To further increase the storage density, the gas storage experiments in slurries with solid content of 40 wt.% were conducted under different chilling temperatures (runs 7~10, 3). As shown in Table 1, the gas uptake decreased with the decrease of chilling temperature. Generally, gas adsorption is more pronounced under lower temperature. The low gas uptake acquired under lower temperature was mainly caused by the freeze of water—the water was easier to freeze under lower temperature, which would weaken the adsorption and hydration by hindering the gas transfer. The highest gas uptake of 133.59 V/V_bed_ was acquired at chilling temperature of 268.15 K, which was higher than that of the slurries and fixed beds at chilling temperatures of 263.15 K (runs 1~6), suggesting the gas uptake could be further improved by adjusting the operation conditions. It was much higher than that in the fixed beds in our previous work (Xiao et al., 2019) and in the suspensions (Kim et al., 2011; Chari et al., 2015). It was noted that in run 9, the gas uptake was 103.93 V/V_bed_, which was slightly lower than that in runs 3, while the gas loss in run 9 was much lower. This was because the lower temperature decreased the dissociation rate of hydrate, leading to less escaped gas during depressurization. The phenomenon that lower temperature leads to smaller gas loss was especially obvious at temperature of 261.15 and 259.15 K when compared with that at 268.15 and 265.15 K.

### Gas Release Rate

**Figure 4:**
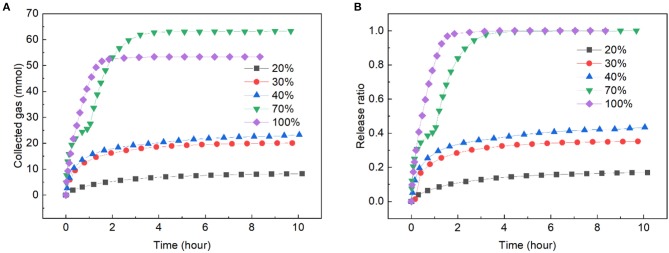
Gas release rate in ZIF-8 slurries and fixed beds with different solid contents at 263.15 K and 0.1 MPa. **(A)** The change of collected gas with time. **(B)** The change of release ratio with time.

A typical hydrate-based gas transportation process includes hydrate formation, transportation and regasification, thus, the gas release rate during hydrate transportation is an important factor to evaluate the transportation method. In runs 1 ~ 6, the gas release rate was investigated in different slurries and fixed beds. The amount of released gas and the gas release ratio over time are shown in Figure 4. Gas release test was not been conducted in run 4 because the gas uptake was too small. As it can be seen in Figure 4A, the gas release rate monotonously increased with the increase of solid content, and the gas released faster in fixed beds than that in slurries. This was because a big part of gas was fixed by adsorption in the fixed beds, while in the slurry, the adsorption was weakened by the existence of large amount of water, and a big part of gas was enclathrated in hydrate. In Figure 4B it could be found that in the dry ZIF-8, all of the gas released within 2 h. That was slower in the fixed bed with solid content of 70 wt.%, however, it was also much faster than that in the slurries. In the slurry with solid content of 20 wt.%, the gas release rate was the slowest, and just 16.9% of the gas released at 10.17 h. In Liang et al. (2005) work, 37% of the methane hydrate dissociated at 4.5 h and 264.4 K in the wet activated carbon fixed bed. When compared with that, the ZIF-8 slurry displayed a slower hydrate dissociation rate than fixed bed.

There existed a very interesting phenomenon during gas releasing in the fixed bed with solid content of 70 wt.%. The curve of collected gas was divided into two parts by an inflection point. Before the point, the released gas was mainly provided by the dissociation of gas hydrate, and after the point it was mainly provided by desorption of the gas. This was because the gas release experiment was performed at 263.15 K, the ZIF-8 particles were wrapped in ice and gas hydrate, and the adsorbed gas was constrained inside the ice though the adsorption equilibrium had been broken after depressurizing the reactor to 0.1 MPa. With the progress of hydrate dissociation, the strength of the hydrate decreased because some water appeared during the conversion of hydrate to ice (Melnikov et al., 2009). Thus, when a certain amount of hydrate dissociated, the gas adsorbed on the ZIF-8 released rapidly, leading to a flection point on the curve.

**Figure 5:**
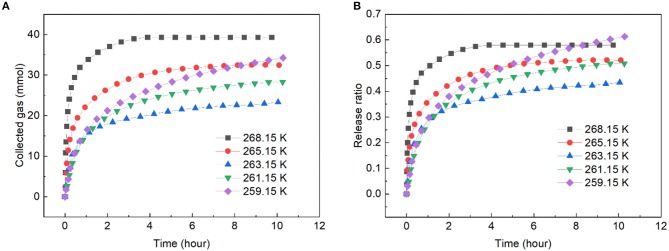
Gas release rate in ZIF-8 slurry with solid content of 40 wt.% at different dissociation temperatures and 0.1 MPa. **(A)** The change of collected gas with time. **(B)** The change of release ratio with time.

Figure 5 presents the gas release from the slurry with solid content of 40 wt.% at different dissociation temperatures under 1 atm. As shown, an obvious self-preservation phenomenon appeared in the slurry. The slowest hydrate dissociation is typically occurred at 268.15 K in pure water. When ZIF-8 was added into the water, that point seemed to have shifted to a lower temperature. This was because the impurity of water and the small hydrate particle would increase the hydrate dissociation (Takeya et al., 2005), and in this study, the addition of ZIF-8 increased the impurity of water and porosity of hydrate. The phenomenon that the shift of the self-preservation temperature window has been reported by Prasad and Kiran (2019). The gas release was the fastest at 268.15 K and the slowest release occurred at 263.15 K. At 268.15 K, 57.98% of the gas released at 5 h, this was very close with that in the carbon fixed bed at 268.15 K in our previous work (Xiao et al., 2019). From Figure 5B, it was noted that at 263.15 K, 43.42% of the gas released at 10 h, which was faster than that in run 1, indicating that decreasing temperature perhaps is not a good choice to retard the gas release.

**Figure 6:**
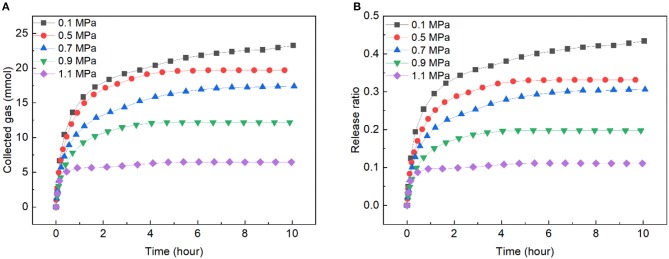
Gas release rate in slurry with solid content of 40 wt.% at different pressures and 263.15 K. **(A)** The change of collected gas with time. **(B)** The change of release ratio with time.

In order to decrease the gas release rate, reducing the driving force by increasing the pressure was adopted. Figure 6 presents the gas release rate of the slurry with solid content of 40 wt.% at 263.15 K and under different pressures. As shown, the release rate decreased with the increase of pressure. At 0.1 MPa, 43.42% of the gas released at 10 h, while only 11.08% of the gas released at 1.1 MPa, which was close to the hydrate dissociation rate in pure water in Liang et al. (2005) work. and it was much slower than the hydrate dissociation in wet carbon bed in Liang et al. (2005) work and our previous work (Xiao et al., 2019). Notably, the gas released with a certain rate at 10 h under 0.1 MPa, while the release ratio of the gas increased very slow under pressure from 0.5 ~ 1.1 MPa at 10 h, and the curves were almost plat after 7 h for these pressure, indicating that even after a long-time transportation under a suitable pressure, large amount of the gas could remain in the frozen bed, and low-pressure transportation vessels could be used in such transportation to reduce the cost.

## Conclusions

An adsorption-hydration sequence method was adopted in water/porous material mixtures in the purpose of hydrate-based gas storage and transportation. The effect of solid content, temperature on the storage capacity, and the effect of solid content, temperature, pressure on the gas release rate were systemically investigated. 37.83 and 98.62% of the adsorption capacity of methane on dry materials remained in activated carbon and ZIF-8 slurries, respectively. In the ZIF-8 fixed beds, when solid content was 60 wt.%, no hydrate formed under quiescent condition. The highest storage density of 127.41 V/V_bed_ was achieved with solid content of 70 wt.%. By the adsorption-hydration sequence method, satisfactory storage capacity could be acquired even in slurries with solid contents of 20 ~ 40 wt.%, and the highest storage density at 263.15 K reached to 107.51 V/V_bed_. The gas loss during depressurization increased with the increase of solid content, and 29.70% of the gas that had already stored in dry ZIF-8 escaped during depressurization. Though the storage density of the fixed bed was higher than that in the slurry, the much lower gas loss during depressurization makes the slurry a good choice for gas transportation. The storage density of the slurry monotonically decreased with the decrease of temperature because under lower temperature water was easier to freeze and then affected the hydrate formation.

In gas release experiments in the fixed beds and slurries, the release rate increased with the increase of solid content, and in dry ZIF-8, all of the gas released within 2 h. In the bed with solid content of 70 wt.%, a two-stage release phenomenon could be found because of the gas adsorption and hydration. To retard the gas release, decreasing the temperature did not acquire a satisfactory result. The self-preservation phenomenon could be found in the slurry, however, even at the temperature that provide the slowest gas release rate, 43.42% of the gas released within 10 h, suggesting under atmospheric pressure, adjusting the temperature could not effectively control the gas release. By increasing the pressure, the gas release was well retarded. The gas release rate decreased with the increase of the pressure. At 1.1 MPa, the release ratio was only 11.08% at 10 h, and from the approximately straight line of the release ratio, it could be inferred that the frozen bed could be stored for a long time. In short, with the adsorption-hydration sequence method, up to 133.59 V/V_bed_ of the storage density could be achieved, and by increasing the pressure to 1.1 MPa, 88.92% of the gas stored in the sample could be kept in the frozen bed, suggesting this method is of great potential for gas storage and transportation.

## Data Availability Statement

All datasets generated for this study are included in the article/supplementary material.

## Author Contributions

PX, G-JC, C-YS, and Q-LM designed the experiment, analyzed the data, and drafted the manuscript. J-LC conducted the experiment. D-XZ, M-KY, and E-BZ helped in preparing figures and calculating data. All authors contributed to the discussions and reviewed the manuscript.

## Conflict of Interest

The authors declare that the research was conducted in the absence of any commercial or financial relationships that could be construed as a potential conflict of interest.
